# A Case of Polypus Arising from the Urethra of a Female, Complicated with Four Bands of Adhesion of the Vagina, and Complete Occlusion of the Os and Cervix Uteri

**Published:** 1869-04

**Authors:** W. H. Williams

**Affiliations:** Louisiana


BUFFALO

### Medical and Surgical Journal.

VOL. VIII. APRIL, 1869. No. 9.

### Original Communications.

ART. I.—*A case of Polypus arising from the Urethra of a Female, complicated with four bands of adhesion of the Vagina, and complete occlusion of the Os and Cervix Uteri.* By W. H. Williams, M. D., *Louisiana.*

Margaret Attaway, (colored,) aged twenty-five years, the mother of three children, was admitted into the Infirmary November 5th, 1868, complaining of inability to retain her urine, and that her monthly sickness was on her nearly all the time, and that she had much pain and a feeling of heaviness in the region of the urino-genital organs. She stated that she had not been able to retain her urine at any time within the last three years, and that her youngest child was about three years old; at the birth of this child she was not attended by either physician or midwife.

At the time this woman was admitted she was very much emaciated; had a haggard and despondent appearance of countenance, walked about and seemed indifferent to all things around her, and would not eat unless forced to do so. We attempted to make a digital examination per vaginum, but found the parts so tender that it was impossible to make a thorough one, and we therefore resorted to chloroform, which enabled us to make a careful examination of the parts, which I will endeavor to describe, as follows:

A polypus, the size of a hen's egg, was found separating the vulva. Pushing this to one side and continuing the finger up the vagina, another and smaller mass than the first was easily felt, and of the same character; and above this again the walls of the vagina were found bound together by bands of organized lymph, which rendered it impossible for the finger to reach the neck of the womb. We had very little difficulty in satisfying ourselves as to the character and origin of the obstructing growths. By pushing the mass lying in the vulva backward and upward into the vagina, three fingers could without difficulty be passed through the urethra into the bladder; in fact the urethra was larger than the vagina itself.

The polypus masses spoken of above were found attached by a common pedicle to the mucous surface, anteriorly to and just at the neck of the bladder. On examination it was found that this pedicle, forked, and was the root of both polypoids. The pedicle was easily divided by introducing two fingers into the urethra and putting the parts on the stretch laterally and cutting it off with a pair of scissors. This done the bleeding surface was touched with the per chlod. of iron, which soon stopped all bleeding, and enabled us to make a thorough examination of the vagina. This was done by using Sim's speculum, which enabled us to divide the bands of attachment from side to side, until they were all removed, and the neck of the womb brought fully into view. The cervix was large, long, and of a glistening appearance, and there was no sign of an os. The bleeding that followed the dividing of the bands was controlled by the iron as above used. The vagina was filled with cotton saturated with the following combination:

Ol Lini, - - - - ℥iiss.
Carbolic Acid, Sim's Solution, - - ℥ss. M.

This tampon was not removed for three days, notwithstanding the pain and restlessness were such as to require the free use of anodynes while it was in situ. The cotton was removed from the vagina on the third day, and was not at all offensive. About the fifth day after the operation the urine had ceased to dribble away, and could be voided at will. Fifteen days later the general condition of the patient was much improved and the soreness about the vulva all gone.

We now determined to try to relieve the occlusion of the cervix; Sim's speculum was again used and the cervix brought plainly to view, which, with a curved volsella, was pulled down a little, and with a tenotome its center pierced to the extent of little over two inches, when it was very evident that the knife had entered the cavity of the uterus. Bilateral incisions were made from this puncture, each way through the os internum and cervix, and a large size sponge tent introduced and retained twenty-four hours, which upon removal was not at all offensive. We thought it best to wait two days before introducing another tent, which was accordingly done and allowed to remain twenty-four hours. In removing this tent there was no trouble in introducing the finger into the womb, and the cervix, external and internal, looked quite healthy. Nothing more was done, nor was it necessary to do anything more in this case. This patient was just two months in the Infirmary, and when discharged was perfectly well.

I will here remark that the way in which I make my sponge tent seems to prevent that bad smell that Dr. J. Marion Sims found so troublesome. My plan may be an old one to some, but I will venture to give it at all events. The combination is this:

| | | |
|---|---|---|
| ℞ | Price's Glycerine, - - - | ℥iv. |
| | Carbolic Acid, (cryst.) - - - | ʒss. |
| | Pulv. Acacia, - - - - | ʒj. M. |

The sponge is to be saturated in this in the same manner as in the mucilage. No bad smell will follow the removal of a tent prepared in this way, even if it remains forty-eight hours, provided a pledget of cotton be dipped in glycerine and introduced into the vagina sufficiently high up to come in contact with the neck of the womb.

Operation performed in presence of and assisted by Drs. Cutliff and Clay, and P. B. Tuzavant and Horace Williams, students.

---

Claret.—No variety of wine is more dangerous to use than what is called *claret*. It is usually a vile mixture. Thousands of gallons are made by allowing water to soak through shavings, and adding thereto a certain proportion of logwood and tartaric acid, and a little alcohol. Good judges can hardly discriminate between this factitious mixture and the genuine article.—*Boston Journal of Chemistry*.

